# Epigenetic regulation of RNA methylations in gastric cancer

**DOI:** 10.3389/or.2025.1601511

**Published:** 2025-11-12

**Authors:** Kaijie Liu, Yafeng Liu, Shujun Zhang, Ziang Li, Wenbing Qu, Penghui Li, Xinjun Hu

**Affiliations:** 1 Department of Infectious Diseases, The First Affiliated Hospital, College of Clinical Medicine, Henan University of Science and Technology, Luoyang, Henan, China; 2 Department of Paediatric, The First Affiliated Hospital, College of Clinical Medicine, Henan University of Science and Technology, Luoyang, Henan, China; 3 Department of Gastrointestinal surgery, The First Affiliated Hospital, College of Clinical Medicine, Henan University of Science and Technology, Luoyang, Henan, China; 4 Henan Medical Key Laboratory of Gastrointestinal Microecology and Hepatology, Luoyang, China

**Keywords:** gastric cancer, epigenetics, RNA methylation, M6A, m5C, m1A, m7G

## Abstract

Gastric cancer (GC) remains a major global health challenge due to its high incidence and mortality. Emerging evidence underscores the critical role of RNA methylation, a key layer of epigenetic regulation, in GC pathogenesis. This review synthesizes current knowledge on various RNA modifications, including m^6^A, m^5^C, m^1^A, and m^7^G, in GC. We critically evaluate the functions of their regulatory proteins (writers, erasers, readers) in modulating oncogenic signaling, metastasis, and tumor immunity. Among these, m^6^A and m^5^C modifications currently present the most compelling evidence, demonstrating significant correlations with patient prognosis and therapy resistance. Furthermore, we explore the translational potential of targeting the RNA methylation machinery, discussing both promising avenues and existing challenges in drug development. This comprehensive analysis aims to provide deeper mechanistic insights and highlight novel therapeutic opportunities for GC.

## 1 Introduction

GC is among the most prevalent malignant tumors worldwide (1). Its high morbidity and poor prognosis render it a significant public health concern. Although advancements in early diagnosis and treatment strategies, particularly through immunotherapy and targeted therapy, have led to improvements in the overall survival rate of patients with GC, the prognosis remains unsatisfactory (2). Recent studies have increasingly highlighted the role of epigenetics in the onset and progression of GC (3). Consequently, there is an urgent need for the identification of effective molecular diagnostic and therapeutic targets, making it crucial to investigate the molecular mechanisms underlying epigenetic modifications associated with the development of GC.

**FIGURE 1:**
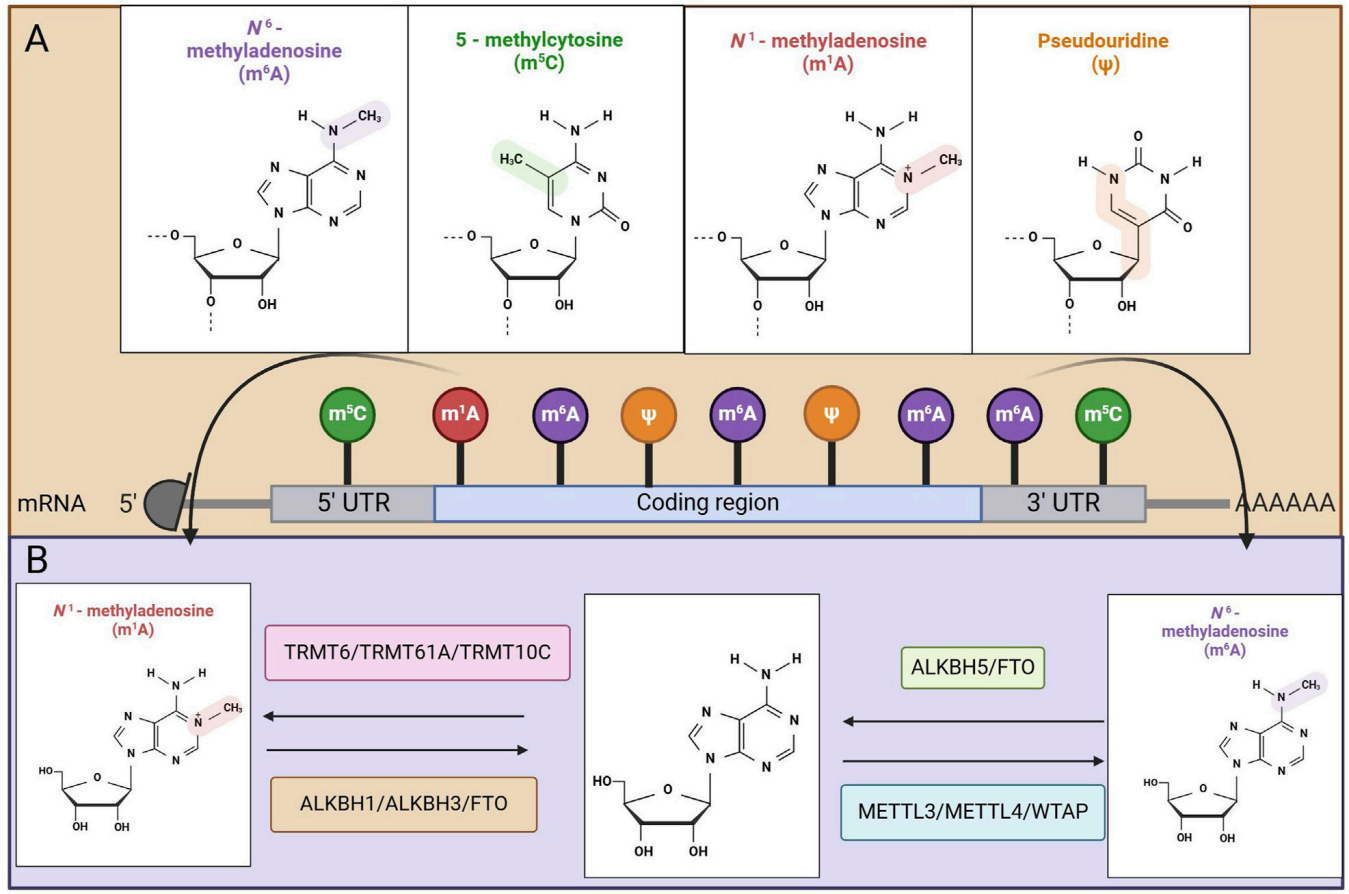
Mapping of RNA modifications machinery. **(A)** Methylation modification in MRNA. **(B)** Mechanisms of action of methyltransferase and demethylase.

Epigenetic modifications play a critical role in the regulation of gene expression and can occur in various biomolecules, including DNA, RNA, and proteins (4). These modifications encompass a range of processes, such as DNA methylation, RNA methylation, and histone modifications. Each of these epigenetic changes contributes to the intricate regulatory mechanisms that control how genes are expressed within an organism (4). In the vast landscape of biological regulation, researchers have identified over 150 distinct types of RNA modifications. These modifications are not exclusive to a single type of RNA but span multiple categories, including noncoding RNAs (ncRNAs) (5). Among the various forms of ncRNA, significant examples include messenger RNA (mRNA), transfer RNA (tRNA), ribosomal RNA (rRNA), small nuclear RNA (snRNA), and small nucleolar RNA (snoRNA). Additionally, other notable forms of ncRNAs, such as microRNAs, long intergenic noncoding RNAs (lincRNAs), and circular RNAs (circRNAs), also exhibit diverse modifications, highlighting the complexity and importance of RNA modifications in gene regulation (5). Recent studies have increasingly focused on the role of RNA methylations in GC, thereby opening new avenues for research in this field. Methylation modifications were first identified in 1974 and include M^6^A, M^5^C, and M^1^A, which rank among the most common and extensively distributed chemical alterations observed in both mRNAs and noncoding RNAs (6) (Figure 1A).

In this review, we provide an overview of the various types of RNA methylations and the corresponding modifying enzymes. Our focus is primarily on elucidating the mechanisms of action of N6-methyladenosine (M^6^A), N1-methyladenosine (M^1^A), 5-methylcytidine (M^5^C), N7-methylguanosine (M^7^G), inosine (I), 2′-O-methylated nucleoside, 3-methylcytidine (M^3^C), and pseudouridine (Ψ) modifying enzymes in the context of GC. Additionally, we explore the applications of RNA methylations in the diagnosis and prognosis of this disease. Finally, we discuss potential treatment strategies for GC that involve RNA methylations. This analysis sought to identify innovative strategies for diagnosing, treating, and predicting outcomes in patients with GC.

## 2 Types of RNA methylations and modifying enzymes

### 2.1 M^6^A

M^6^A is the leading type of RNA methylation found in eukaryotic organisms and is the most abundant internal modification of mRNA, occurring primarily within the conserved sequence RRACH (where R represents either G or A and can also be C or U) (7). The process of M^6^A modification involves chemical modification of the sixth nitrogen atom in RNA, which is mediated by regulatory proteins known as writers, erasers, and readers (8). Currently, M^6^A RNA methylation plays a critical role in nearly all stages of mRNA regulation, including transcription (9), splicing (10), stability maintenance (11), and translation (12), establishing a broad range of regulatory mechanisms for gene expression (13). M^6^A methylation is characterized as a dynamic and reversible chemical modification process that involves three types of molecules: methyltransferases that identify methylation binding sites (commonly referred to as M^6^A writing proteins), demethylases that target demethylation sites (known as M^6^A erasing proteins), and methylation recognition proteins (termed M^6^A reading proteins) (14) (Figure 1B). The following section introduces the roles of these writers, erasers, and readers.

#### 2.1.1 M^6^A writers

There are three primary methylases involved in M^6^A methylation: methyltransferase-like 3 (METTL3), methyltransferase-like 14 (METTL14), and nephroblastoma-1 associated protein (WTAP) (15). METTL3 is an S-adenosylmethionine (SAM)-binding protein; as the core component of the M^6^A methyltransferase complex (MTC), it is highly conserved across eukaryotes from yeast to humans (16). METTL14 is another catalytic component of the M^6^A MTC. METTL3 and METTL14 co-localize in nuclear speckles and form a stable heterodimer with a 1:1 stoichiometry, functioning as the catalytic core of the complex (17). However, only METTL3 functions catalytically, utilizing its internal SAM-binding domain to transfer the methyl group from SAM to adenine bases in RNA, generating S-adenosylhomocysteine (SAH) as a by-product. In contrast, METTL14 primarily functions to stabilize the structure of the MTC. WTAP lacks catalytic activity and primarily facilitates M^6^A installation by recruiting METTL3 and METTL14 to nuclear speckles (18). RNA-binding motif protein 15 (RBM15) and its paralog RBM15B lack catalytic activity but facilitate M^6^A modification by binding to both METTL3 and WTAP, subsequently recruiting this complex to specific RNA sites (19). Furthermore, ZC3H13 (zinc finger CCCH-type containing 13) possesses an amino acid sequence in which approximately 80% comprises low-complexity (LC) domains. These LC domains may facilitate its targeting to nuclear speckles. Following its direct interaction with WTAP, ZC3H13 anchors the MTC within nuclear speckles via its LC domains, thereby enhancing the complex’s catalytic efficiency. Unlike METTL3, other components of the catalytic complex lack RNA methyltransferase activity. Methyltransferase-like 16 (METTL16) is a newly identified, independent RNA methyltransferase that catalyzes M^6^A deposition at specific sites, including within the 3′UTRs of certain mRNAs and at position A43 of U6 snRNA (20). Furthermore, the potential existence of additional, less explored M^6^A methyltransferases remains a plausible consideration.

METTL3 is an essential methyltransferase distinguished by its characteristic methyltransferase domain. This specific domain is integral to its ability to recognize substrate RNA effectively. Once the substrate RNA is identified, METTL3 catalyzes the transfer of a methyl group from its typical donor, which is most commonly SAM (16). This chemical process occurs at the N6 position of adenine (A) within the RNA molecule, leading to the M^6^A modification. The structural properties of METTL3 facilitate its interaction with other proteins, thereby playing a pivotal role in the M^6^A modification process. As a core component of the M^6^A MTC, METTL3 collaborates with other proteins, such as METTL14, to modify various types of RNA through the addition of M^6^A. For example, in messenger RNA (mRNA), METTL3-mediated M^6^A modification influences splicing, transport, translation, and stability (16). This increased expression of METTL3 enhances the translation of specific oncogene mRNAs via M^6^A modification, subsequently promoting the proliferation and survival of tumor cells. These findings suggest that METTL3 may serve as a potential target for cancer therapy, as it is significantly overexpressed in lung cancer, liver cancer, and GC and plays a role in regulating tumor progression (22–24).

METTL14 contains domains associated with RNA binding and methyltransferase activity (16). Its structure can closely associate with METTL3 to form a heterodimer (18). Although the methyltransferase activity of METTL14 is relatively weak, it provides a stable RNA binding platform for METTL3, enhancing the recognition and binding capacity of the entire complex to RNA substrates (16). The synergistic interaction between METTL14 and METTL3 is a crucial component of the M^6^A methyltransferase complex. During RNA methylation, METTL14 aids in locating the substrate RNA, allowing METTL3 to more accurately add methyl groups to specific adenine residues. In terms of gene expression regulation, METTL14-mediated M^6^A modification can also influence the metabolic processes of mRNAs. For example, during neural development, METTL14 plays a role in regulating the M^6^A modification of mRNAs related to nerve genes, thereby impacting the differentiation of neural stem cells and the maturation of neurons (25).

WTAP is a crucial component of the M^6^A methyltransferase complex. Although it lacks direct catalytic activity, it plays a significant role in the assembly and localization of the complex (18). WTAP contains multiple protein interaction domains that facilitate interactions with METTL3, METTL14, and other subunits, thereby forming a stable complex and positioning it at the transcription site within the nucleus. By influencing the location and stability of the complex, WTAP indirectly regulates the level of M^6^A modification, which in turn affects the activity of the M^6^A methyltransferase and the accessibility of RNA substrates (18). During cell differentiation, WTAP is involved in modulating the M^6^A modification of related gene mRNAs, ensuring normal cellular differentiation. Research has shown that WTAP is abnormally expressed in various tumors and is correlated with tumor malignancy and patient prognosis. For example, in hepatocellular carcinoma, the upregulation of WTAP expression may influence the level of M^6^A modification, promoting the proliferation, invasion, and metastasis of hepatocellular carcinoma cells (26).

#### 2.1.2 M^6^A erasers

In addition to methyltransferases, the presence of M^6^A demethylases imparts unique dynamic and reversible properties to M^6^A methylation (27). Currently, two primary M^6^A demethylases are recognized: fat mass and obesity-related protein (FTO) and AlkB homolog 5 (ALKBH5) (28,29). FTO and ALKBH5 belong to the α-ketoglutarate (α-KG)-dependent dioxygenase family. They catalyze M^6^A demethylation in an Fe(II)- and α-ketoglutarate-dependent manner. Additionally, recent studies have identified AlkB homolog 3 (ALKBH3) as another m6A demethylase (30).

FTO is a dioxygenase that relies on Fe(II) and α-ketoglutarate (α-KG) for its activity. It possesses a typical dioxygenase domain, with an active site capable of binding Fe(II) ions. α-KG serves as an auxiliary factor in the reaction, enabling FTO to recognize and catalyze the demethylation of M^6^A (31). Research has confirmed that FTO can convert M^6^A in RNA to N6-hydroxymethyladenosine and N6-formyladenosine through a stepwise oxidation process (32). FTO is involved in various aspects of mRNA metabolism, including the removal of M^6^A modifications, which influence mRNA splicing and stability (33). As the first identified demethylase, FTO has also been implicated in tumorigenesis, including in conditions such as melanoma, ovarian cancer, and renal cell carcinoma (34–36).

ALKBH5 is a significant member of the AlkB family and is known for its unique enzymatic activity (37). ALKBH5 is classified within the α-KG/Fe(II)-dependent dioxygenase family, and its structure includes a highly conserved dioxygenase domain that specifically recognizes M^6^A-modified RNA substrates, facilitating the catalytic demethylation of M^6^A through the action of Fe(II) and α-KG (31). ALKBH5 plays a key role in the modification of mRNAs after transcription, affecting processes such as mRNA nucleation, transport, and subsequent translation (38). Increasing evidence suggests that ALKBH5 plays a significant role in the progression of GC. The demethylase ALKBH5 enhances the viability of GC cells by lowering the M^6^A levels of certain genes and suppressing the expression of genes associated with apoptosis (39). For example, the demethylase activity of ALKBH5 has been shown to inhibit the invasion of GC cells through the M^6^A modification of PKMYT1 (38).

#### 2.1.3 M^6^A readers

In addition to the dynamic regulation of M^6^A methyltransferases and demethylases, M^6^A-binding proteins must specifically recognize M^6^A modifications to influence subsequent biological functions (7). These M^6^A-binding proteins are functionally analogous to ‘readers’ and can be categorized into three main groups. Proteins that contain YT521-B homologous (YTH) domains represent the primary category of M^6^A readers, as they directly interact with M^6^A-methylated RNA to mediate its functions (40). Concurrently, the heterogeneous ribonucleoprotein (HNRNP) family, which includes HNRNPA2B1, HNRNPC, and HNRNPG, is classified as indirect M^6^A readers (41). These proteins bind to RNA at sites that undergo structural alterations due to M^6^A methylation rather than directly recognizing M^6^A sites. Additionally, insulin-like growth factor 2 mRNA-binding proteins 1, 2, and 3 (IGF2BP1/2/3) function as M^6^A readers, playing crucial roles in the stability of M^6^A-containing RNA (42).

The recognition and aggregation of M^6^A-modified mRNAs lead to the recruitment of associated degradation enzymes, thereby facilitating mRNA degradation and regulating its stability and lifespan (43). The IGF2BP protein family specifically recognizes and binds to M^6^A-modified mRNAs, primarily by targeting the 5′untranslated region or the 3′untranslated region of the mRNA. This binding stabilizes the mRNA, preventing its degradation and consequently increasing both its half-life and expression level. In tumor cells, the expression of the IGF2BP protein family is frequently upregulated, which enhances the expression of tumor-related genes and contributes to tumorigenesis and progression (44).

The interaction between M^6^A methyltransferases and demethylases governs the reversible regulation of M^6^A abundance and distribution in RNA. Additionally, M^6^A-binding proteins recognize and interact with M^6^A, facilitating a range of biological functions, including RNA processing, transport, and translation (45).

### 2.2 M^5^C

In addition to the M^6^A methylation, cytosine methylation and the formation of M^5^C at the C^5^ site, which was first discovered in *Escherichia coli* in 1958, represent another significant RNA modification (46). 5-Methylcytosine is a prevalent RNA modification that influences cellular function by regulating protein expression (47). Like the M^6^A modification, the M^5^C modification is reversible and can be regulated by writer proteins, reader proteins, and eraser proteins. While M^5^C modifications are found in smaller quantities than M^6^A modifications, which represent approximately 1% of the total adenine nucleotides, M^5^C modifications make up approximately 0.02% of all cytosine nucleotides (48). As interest in the M^5^C modification of mRNAs has increased, several regulatory factors have been identified, including the M^5^C methyltransferases (writers) NSUN2 and NSUN6, as well as the demethylases (erasers) TET2 and ALKBH1. Currently, known readers of M^5^C include ALYREF, YBX1, YBX2, YTHDF2, RAD52, and FMRP (49) (Figure 1B). The following section provides an introduction to these writers, erasers, and readers.

#### 2.2.1 M^5^C writers

RNA M^5^C methyltransferases typically possess a catalytic domain that consists of a structural core of approximately 270 amino acids, alongside a binding site for S-adenosylmethionine (50). In mammals, the introduction of the M^5^C modification is catalyzed by members of the DNMT2 and NSUN families (NSUN1--NSUN7) (51).

NSUN2 is implicated in promoting cell proliferation by destabilizing mRNA, a process that relies on a specific mechanism involving M^5^C (52). NSUN2 is found mainly in the nucleus or in both the nucleus and the cytoplasm, indicating that it might play a role in different biological functions depending on where it is located within the cell (53). Throughout the various phases of the cell cycle, the localization of NSUN2 significantly changes, transitioning from the nucleus to the mitotic spindle (54). Additionally, as an M^5^C methyltransferase, NSUN2 predominantly exhibits methyltransferase activity within the nuclear environment (55). In contrast, NSUN5 functions as a eukaryotic ribosomal RNA methyltransferase that specifically binds to cytosine in 28S rRNA to facilitate protein translation (56). Research has demonstrated that elevated expression levels of NSUN5 in glioma cells contribute to dysregulated protein synthesis and tumor proliferation (57).

#### 2.2.2 M^5^C erasers

The erasers modified by M^5^C include primarily members of the TET family and ALKBH1. The TET family comprises TET1, TET2, and TET3, all of which are known as demethylases for DNA (58). These proteins play a role in the methylation of tRNA, thereby impacting tRNA translation. The hydroxymethylation of RNA mediated by TET proteins reduces essential pluripotency factors, thus improving transcript stability and significantly contributing to various biological processes, such as the regulation of gene expression and tumor development (59). The dioxygenase ALKBH1 promotes the transformation of M^5^C into hM^5^C and 5-formylcytidine (f^5^C), specifically at the C34 position of tRNA in both cytoplasmic and mitochondrial locations. The function of this enzyme has been shown to influence mitochondrial activity by decreasing translation and oxygen usage (60). Consequently, the conversion of M^5^C to hM^5^C results in a decrease in the overall level of M^5^C modification. Thus, both TETs and ALKBH1 are key proteins involved in the erasure of RNA M^5^C methylation.

#### 2.2.3 M^5^C readers

The primary modified readers of M^5^C include ALYREF, YBX1, YBX2, fragile X mental retardation protein (FMRP), and splicing factor 2 (SRSF2), all of which are abundant in serine/arginine (49). ALYREF possesses a classic RNA binding motif that predominantly interacts with the 5′and 3′regions involved in mRNA export (61). It facilitates the transport of M^5^C-modified RNA from the cytoplasm to the nucleus while maintaining its stability and is implicated in the development of various malignant tumors. YBX1 is a versatile protein that features a cold shock domain (CSD), which has been preserved throughout evolution and is classified within the group of RNA binding proteins (RBPs). This protein is involved in both transcription and translation processes and functions as a splicing factor (62).

### 2.3 M^1^A

M1A appears on the first nitrogen atom of adenosine in RNA and is a ubiquitous RNA modification first reported in 1961 (63). M1A was subsequently shown to be present in tRNAs (64), rRNAs (65), mRNAs (66), and long-chain noncoding RNAs (lncRNAs) (67). Like the dynamic modification of M^6^A, M1A is added by methyltransferases, referred to as writers; removed by demethylases (ALKBH1, ALKBH3, ALKBH7, FTO), known as erasers; and recognized by M^1^A-binding proteins (YTHDF1, YTHDF2, YTHDF3, YTHDC1), termed readers (68). The following is an introduction to writers, erasers, and readers.

#### 2.3.1 M^1^A writers

The M^1^A methyltransferase complex primarily comprises TRMT6, TRMT61A, and TRMT10C. TRMT61A features a binding pocket for the methyl donor SAM and serves as the catalytic subunit. Together, TRMT61A and TRMT6 form a functional heterotetramer complex (69). Notably, TRMT6 does not possess a SAM-binding motif, which is crucial for tRNA binding. The TRMT6/61A complex is localized in the cytosol and appears to recognize its target in a structure-dependent manner. Additionally, TRMT10C is responsible for installing mt-tRNA M^1^A and/or M^1^G at position 9, which may be associated with SDR5C1 (70).

#### 2.3.2 M^1^A erasers

The erasers of M^1^A primarily include ALKBH1, ALKBH3, and FTO. ALKBH1 is a 2-oxoglutarate-and Fe2+-dependent dioxygenase with multiple cellular functions. Studies have found that the frequency of M^1^A in two mitochondrial tRNAs increased in ALKBH1 knockout cells, indicating that ALKBH1 has demethylation activity on M^1^A in mitochondrial tRNAs (71). ALKBH3 is responsible for demethylating both M^1^A and M3C in tRNA, as well as M^1^A in mRNA (72). FTO was the first identified RNA m^6^A demethylase and is known to bind and demethylate a variety of RNA species, including M^6^A and M^6^Am in mRNAs and snRNAs, as well as M^1^A in tRNAs (73).

#### 2.3.3 M^1^A readers

Compared with studies focused on ‘writers’ and ‘erasers’ of M^1^A, research on ‘readers’ remains relatively scarce. Recent findings have identified four proteins containing YT521-B homologous (YTH) domains—YTHDF1, YTHDF2, YTHDF3, and YTHDC1—as M^1^A-modified readers (74). These proteins can directly bind to RNA with M^1^A modifications; however, their binding affinity for M^1^A sites is weaker than that for M^6^A. The YTH domain is present in 174 different proteins and is evolutionarily conserved across eukaryotic species, suggesting that the YTH domain is a critical structural element for recognizing methyl groups in RNA (75). Nonetheless, research into the functional roles of these proteins as M^1^A readers remains limited.

### 2.4 M^7^G

M^7^G is a modification wherein a methyl group is added to the seventh nitrogen atom of RNA guanine (G) by the action of methyltransferases (76). This modification represents one of the most prevalent forms of base alteration in post-transcriptional regulation. M^7^G is recognized as the most abundant tRNA modification, primarily catalyzed by the methyltransferase-like 1 (METTL1)/WD repeat domain 4 (WDR4) complex within the variable loop of tRNAs. In this methyltransferase complex, METTL1 serves as the catalytic subunit for M^7^G deposition, while WDR4 plays a stabilizing role (77). M^7^G is widely distributed in tRNAs, rRNAs, and the 5′cap of eukaryotic mRNAs, where it plays crucial roles in RNA processing, metabolism, stability, nucleation, and translation (76). Previous studies have revealed that M^7^GtRNA modifications can enhance the translation of oncogenic mRNAs and promote the progression of intrahepatic cholangiocarcinoma (78). Analogous to the dynamic nature of the M^6^A modification, M^7^G is installed by methyltransferases known as “writers,” removed by demethylases or “erasers,” and recognized by specific M^7^G-binding proteins, or “readers.” The following section provides an introduction to its associated writers, erasers, and readers.

#### 2.4.1 M^7^G writers

The catalytic process of M^7^G RNA modification is primarily executed by two conserved methyltransferase complexes: the METTL1/WDR4 complex, which modifies specific tRNAs and certain microRNAs, and the RNMT/RAM complex, which catalyzes the formation of the M^7^G cap on messenger RNAs. Accumulating evidence from recent studies confirms that dysregulation of M^7^G modification on tRNAs is closely linked to the pathogenesis of various human diseases (79). Notably, dysfunction of the METTL1 gene or its functional complex with WDR4 has been demonstrated to promote the initiation and progression of multiple malignancies, including lung cancer (80), esophageal squamous cell carcinoma (81), and intrahepatic cholangiocarcinoma (78). However, the precise biological functions and regulatory mechanisms of METTL1-mediated M^7^G modification on genomic DNA, particularly within CpG island sequences, as well as on tRNAs, remain to be fully elucidated.

#### 2.4.2 M^7^G erasers

In epigenetic regulation, dynamically reversible modifications typically require specific demethylases to perform the erasure function. However, for the M^7^G RNA modification, enzymes capable of catalyzing its demethylation with high efficiency and specificity have not yet been unequivocally identified or functionally validated within the scientific community. The absence of this key catalytic component represents a significant gap in the current understanding of the dynamic regulation of M^7^G modification.

#### 2.4.3 M^7^G readers

The biological functions mediated by M^7^G modification depend on specific recognition proteins, known as “readers.” Currently identified M^7^G readers primarily include the following protein families: the eukaryotic initiation factor eIF4E, which recognizes the M^7^G cap of mRNAs to initiate translation; the NCBP1 and NCBP2 subunits that form the nuclear cap-binding complex (CBC) and recognize capped RNAs during nuclear processing; and the QKI protein family (QKI5, QKI6, QKI7), whose members have been reported to potentially recognize internal M^7^G modifications in specific RNA types, thereby regulating RNA metabolism and function.

## 3 The mechanism of RNA methylation in GC

The ability of RNA methylations to promote the proliferation of GC cells and enhance their migratory and invasive capabilities is well documented. These modifications play crucial roles in regulating gene expression. In GC cells, alterations in the RNA methylation status of specific key genes can result in their upregulation. Furthermore, the cytoskeletal proteins encoded by methylated mRNAs can influence the morphology and motility of these cells. This discussion focuses primarily on the mechanisms of RNA methylations in GC, specifically examining M^6^A, M^5^C, and M^1^A modifications, along with their associated enzymes (Table 1).

**TABLE 1:** The mechanism of action of RNA methylation modification in gastric cancer.

Methylation type	Type	Factor	Expression	Mechanism/Pathway	Biological function	Related genes	References
M6A	Writers	METTL 3	Elevated	HBXIP/METTL3/MYC	Promote the proliferation, migration and invasion	MYC、HBXIP	(84)
				Inhibits SOCS2 expression; affects JAK/STAT pathway	Apoptosis, proliferation, metastasis and inflammation	SOCS2	(88)
				METTL3/ZMYM1/E-cadherin; EMT	Promote the migration and invasion	ZMYM1	(24)
				METTL3-ANGPTL3	Promote the proliferation and migration	ANGPTL3	(94)
		METTL 14	Downward	circORC5/miR-30c-2-3p	Promote the proliferation, migration and invasion; Promote tumor progression	circORC5; miR-30c-2-3p	(95)
			Downward	PI3K/AKT/mTOR	Promote the proliferation and invasion	PI3K/AKT/mTOR	(97)
		WTAP	Elevated	Enhances HK2 mRNA stability by binding to the 3′-UTRm6A site	Promote the proliferation	HK2	(100)
			Downward	FAM83H-AS1	Promote the proliferation, migration and invasion	FAM83H-AS1	(101)
		ZC3H13	Elevated	ZC3H13/DLX6-AS1	Promote the proliferation, migration and invasion	ZC3H13	(103)
		RBM15	Elevated	RBM15/IGF2BP2	Promote the proliferation, migration and invasion	IGF2BP2	(105)
	Erasers	FTO	Elevated	circFAM192A/SLC7A5	Promote the proliferation	SLC7A5	(106)
			Elevated	Cag A + *H. pylori*/FTO/CD44	Promote the proliferation, migration and invasion	CD44	(109)
			Elevated	ITGB1-FAK	Promote the migration and invasion	ITGB1	(111)
			Elevated	Not detailed	Promote the migration and invasion	MOXD1	(113)
			Elevated	Deregulation of mitochondrial fission/fusion and metabolism	Promote the proliferation and invasion	Caveolin-1	(115)
			Elevated	SP1-AURKB-ATM	Promote the proliferation, and migration	SP1; AURKB	(123)
			Elevated	FTO-FOS-IGF2BP1/2	Inhibit the invasion in EBVaGC	FOS	(125)
		ALKBH5	Downward	M6A/YY1/ATG4B	Promote the proliferation, and migration	YY1	(127)
			Elevated	ALKBH5/circFOXP1/miR-338-3p/SOX4	Promote the proliferation and invasion	circFOXP1	(131)
			Elevated	ALKBH5/JAK1/STAT3	Promote the proliferation and migration	JAK1	(133)
			Elevated	ALKBH5/NEAT1/EZH2	Promote migration and invasion	NEAT1	(137)
	Readers	YTHDF 1	Elevated	YTHDF1-USP14	Promote the proliferation and migration	USP14	(143)
			Elevated	MNU/YTHDF1/HSPH1	Promote the proliferation	HSPH1	(145)
		YTHDF2	Downward	YTHDF2/PPP2CA	Promote the proliferation and migration	PPP2CA	(150)
		IGF2BP	Elevated	IGF2BP2/CSF2/Notch1	Promote the proliferation and migration	CSF2	(154)
M5C	Writers	NSUN2	Elevated	SUMOylation-NSUN2-m5C	Promote the proliferation and migration	SUMO-2/3	(158)
		NSUN5	Elevated	WNT/β-catenin	Promote the proliferation, migration and invasion	WNT	(160)
	Readers	YBX1	Elevated	HOXC-AS3	Promote the proliferation, migration and invasion	HOXC-AS3	(163)
M7G	Writers	METTL1	Elevated	METTL1/M7G/SDHAF4/SDHA/SDHB/ETC	Promote the proliferation, migration and invasion	SDHAF4	(165)

### 3.1 The role of M^6^A modification in GC

M^6^A modification is vital to many biological processes, especially in relation to the development and progression of various cancers (82). Recent studies suggest that M^6^A modification could play a role in the initiation and progression of tumors (83). In this review, we discuss the role of M^6^A modification in GC, focusing on the functions of M^6^A modification enzymes.

#### 3.1.1 METTL3

METTL3-mediated M^6^A modification enhances cell proliferation, migration, invasion, and apoptosis by promoting the expression of certain oncogenes, including MYC (84). Among these biomarkers, MYC serves as a fundamental oncogene that can induce tumor cell growth and inhibit apoptosis across various cancers (85). In GC, the level of MYC expression is regarded as an unfavorable prognostic indicator for individuals diagnosed with GC (86). Hepatitis B X-interacting protein (HBXIP), located in lysosomes, is known to significantly increase METTL3 expression, contributing to poor prognosis in patients with GC (87). METTL3 expression is upregulated by HBXIP, which results in a sustained increase in the level of the M^6^A modification of MYC mRNA. The reduction in the M^6^A level of MYC mRNA occurs via the inhibition of HBXIP, which interrupts the METTL3-mediated M^6^A regulatory process, thereby ultimately impeding the proliferation, migration, and invasion of GC cells (84).

The upregulation of METTL3 in GC may sustain the carcinogenicity of this disease by inhibiting SOCS2 and promoting cell proliferation (88). SOCS2 is well established as a negative feedback regulator in various proliferation-related pathways and may function as a tumor suppressor gene across multiple malignant tumors (89). SOCS2 can be activated through tyrosine phosphorylation and may serve as a downstream factor in the JAK/STAT pathway, providing negative feedback regulation for this signaling cascade (90). The JAK/STAT signaling pathway is often dysregulated in GC and is essential for several processes in cancer cells, such as apoptosis, proliferation, metastasis, and inflammation (91).

METTL3 is essential for epithelial‒mesenchymal transformation (EMT) in tumor cells *in vitro* and for metastasis *in vivo* (24). Tumor recurrence and metastasis are the primary causes of mortality in patients with GC (92). Furthermore, this study identified zinc finger MYM type 1 (ZMYM1) as an M^6^A target of METTL3, with METTL3-mediated M^6^A modification enhancing the stability of ZMYM1 mRNA. ZMYM1, a member of the MYM protein family, has the capacity to either activate or inhibit transcription (93). It promotes EMT programming and transition by recruiting the CtBP/LSD1/CoREST complex, which mediates the repression of the E-cadherin promoter. These results suggest that M^6^A modification is crucial in GC, emphasizing the METTL3/ZMYM1/E-cadherin signaling pathway as a promising therapeutic target for approaches focused on reducing metastasis and invasion in GC (24).

METTL3 may also play a carcinogenic role in STomach ADenocarcinoma (STAD) by downregulating the expression of ANGPTL3 through an M^6^A-dependent mechanism. Elucidation of the METTL3-ANGPTL3 axis and its impact on STAD tumor growth will enhance our understanding of the mechanisms underlying gastric adenocarcinoma development (94). In this investigation, the coexpression of ANGPTL3 and METTL3 in STAD was analyzed via the TCGA dataset. Lower levels of ANGPTL3 correlated with increased METTL3 levels in STAD samples, which was associated with a reduction in the survival time of patients with STAD. Additionally, ANGPTL3 enrichment was found to inhibit the growth and metastasis of STAD cells. Researchers have also reported that METTL3-mediated M^6^A modification can decrease ANGPTL3 mRNA levels and that the inhibitory effects of METTL3 silencing on cancer progression can be partially reversed by ANGPTL3 inhibition.

**FIGURE 2:**
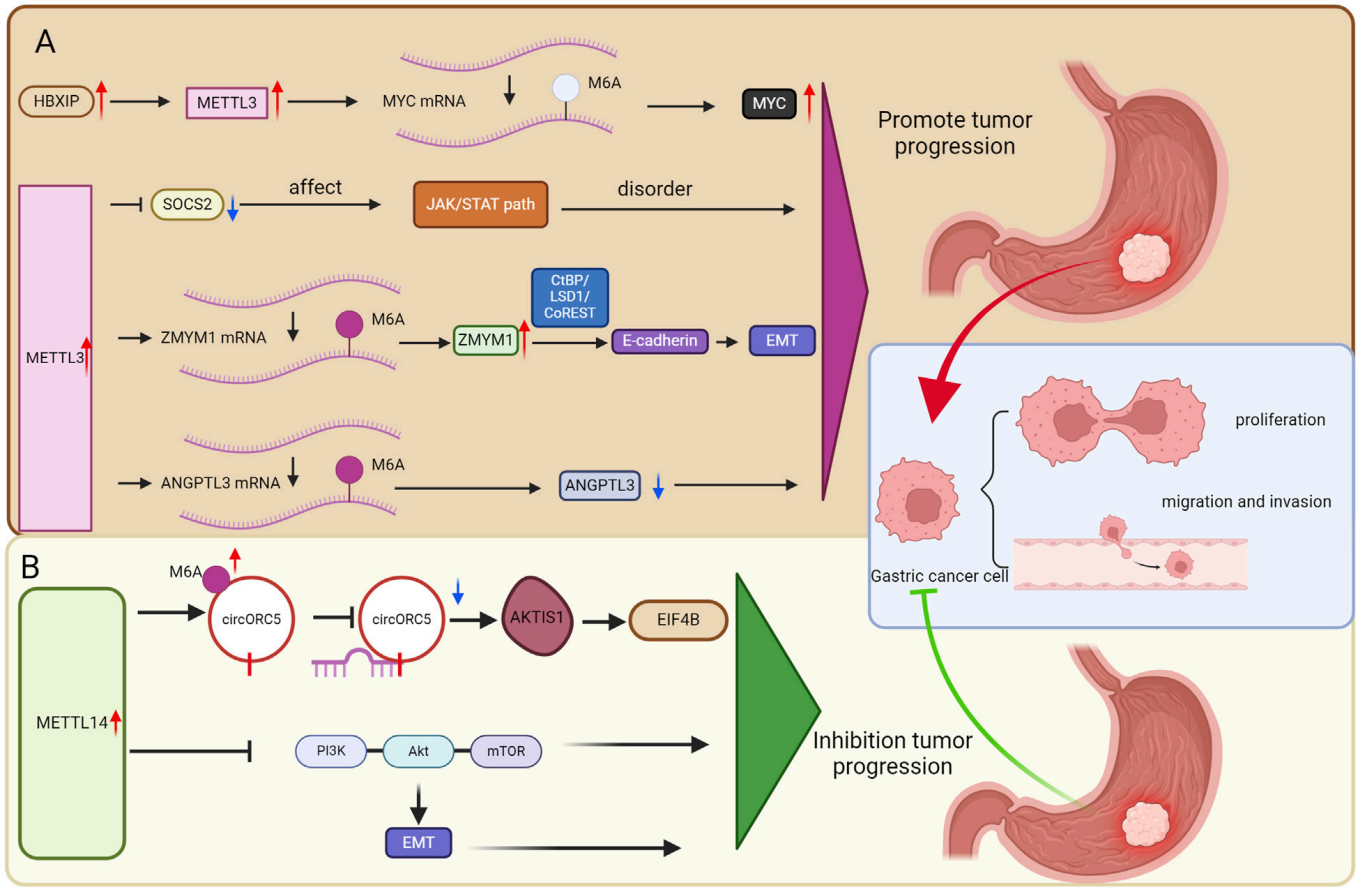
The role of m6a methyltransferase in GC. **(A)** METTL3 as a potential biological target may play a crucial role in GC. **(B)** METTL14 as a potential biological target may play a crucial role in GC.

These findings suggest that METTL3 may serve as a potential biological target and could play a significant role in GC (Figure 2A).

#### 3.1.2 METTL14

METTL14 expression is significantly reduced in GC patients and that decreased METTL14 expression is an indicator of unfavorable survival rates (95). The downregulation of METTL14 resulted in decreased M^6^A levels of circORC5, alongside an increase in circORC5 expression, indicating that METTL14 may exert its influence through M^6^A-dependent modifications of circORC5 (95). CircORC5 can bind to miR-30c-2-3p, which leads to a decrease in its expression in GC cells. Earlier studies have shown that miR-30c-2-3p is reduced in breast cancer, where it plays a role in suppressing cell growth and progression through the cell cycle (96). These results indicate that METTL14-mediated circORC5 promotes the growth of GC by acting as a sponge for miR-30c-2-3p (95). Furthermore, AKT1S1 and EIF4B have been recognized as direct targets of miR-30c-2-3p within these cells (95). These findings suggest that METTL14 suppresses GC progression by enhancing the M^6^A modification of circORC5 while diminishing its expression. Additionally, circORC5 acts as a sponge for miR-30c-2-3p, leading to the upregulation of ElF4B and AKT1S1, which in turn promotes tumorigenesis in GC. In conclusion, the growth and invasion of GC are inhibited by METTL14 through its regulation of the circORC5/miR-30c-2-3p pathway, highlighting METTL14 as a promising therapeutic target for GC (95).

METTL14 can inhibit tumor proliferation and invasion by modulating the PI3K/AKT/mTOR signaling pathway (97). This pathway is a crucial regulatory mechanism for cell proliferation and metabolism, and it is closely associated with tumor initiation and progression (98). Specifically, upon the activation of receptor tyrosine kinases (RTKs) and G protein-coupled receptors (GPCRs), PI3K catalyzes the phosphorylation of phosphatidylinositol 4,5-bisphosphate (PIP2) to produce phosphatidylinositol 3,4,5-trisphosphate (PIP3). Activated PIP3 subsequently recruits and activates AKT in the cytoplasm, which influences the levels of transcription factors involved in EMT and activates matrix metalloproteinases, thereby promoting cell invasion and metastasis (99). In summary, METTL14 serves as a principal regulator of aberrant M^6^A modifications in GC. By targeting the PI3K/AKT/mTOR and EMT pathways as tumor suppressors, METTL14 can inhibit the progression and invasiveness of GC cells.

METTL14 primarily functions as a “brake mechanism” in GC. Its deficient expression leads to diminished suppression of various cancer-promoting RNAs and signaling pathways, thereby effectively “lifting restraints” on tumor development. These findings suggest that METTL14 may represent a significant biological target in the context of GC (Figure 2B).

#### 3.1.3 WTAP

Research has shown that WTAP increases the stability of HK2 mRNA through its interaction with the M^6^A site in the 3′-UTR, which in turn affects the Warburg effect in GC and promotes cancer progression (100). The expression levels of WTAP and M^6^A were elevated in GC tissues and cells, and high WTAP expression was significantly associated with poor prognosis in patients with GC. Additional experiments indicated that WTAP promotes proliferation and glycolysis (including glucose uptake, lactic acid production, and extracellular acidification) *in vitro*, whereas WTAP knockout inhibits tumor growth *in vivo*. Additionally, studies revealed that HK2 is a target of WTAP, confirming that WTAP improves the stability of HK2 mRNA through its interaction with the M^6^A site in the 3′-UTR, thereby influencing the response to the Warburg effect in GC (100).

Another study indicated that WTAP can promote the development of GC by mediating FAM83H-AS1 through M^6^A modification (101). This research demonstrated that WTAP mediates FAM83H-AS1 expression in an M^6^A-dependent manner and that silencing WTAP reverses the carcinogenic effects of FAM83H-AS1 overexpression on the migration, proliferation, and invasion of GC cells (102). This research underscores WTAP as a potential biological target that could be significant in the development of GC.

#### 3.1.4 ZC3H13

Emerging evidence indicates that Zinc Finger CCCH-Type Containing 13 (ZC3H13), a core component of the M^6^A methyltransferase complex, exhibits tumor-suppressive functions in cancer progression (103). Recent studies have detected elevated levels of Zinc Finger CCCH-Type Containing 13 (ZC3H13) in GC samples. ZC3H13 silencing significantly suppressed the proliferation, migration, and invasion of GC cells. Concurrently, it potentiated the inhibitory effects on malignant behaviors in GC cells. Furthermore, this study demonstrated that ZC3H13 suppresses the upregulation of DLX6-AS1 expression in GC cells by mediating M^6^A modification of this lncRNA, consequently destabilizing DLX6-AS1. Collectively, ZC3H13 knockdown potentiates the suppression of malignant phenotypes in GC cells through M^6^A-mediated regulation of DLX6-AS1 (104).

#### 3.1.5 RBM15

A recent study observed significant upregulation of RBM15 in GC, with elevated expression correlating with poor prognosis. RBM15 was found to promote GC cell proliferation and invasiveness. Notably, ATP-citrate lyase (ACLY) was identified as a downstream oncogenic target of RBM15 in GC cells. Mechanistically, RBM15 activates ACLY through IGF2BP2-dependent M^6^A modification, thereby driving lipogenesis and exacerbating malignant phenotypes in GC. This RBM15/IGF2BP2-mediated M^6^A modification promotes ACLY activation, which fuels lipogenesis and accelerates GC progression (105).

#### 3.1.6 FTO

Recent studies have confirmed the carcinogenic role of FTO in GC and explored its underlying mechanisms (106). This research demonstrated that the M^6^A demethylase FTO contributes to the proliferation of GC cells. The mechanism underlying this effect involves FTO binding to specific sites on circFAM192A, leading to the demethylation of the M^6^A modification on circFAM192A. The removal of this modification enhances the stability of circFAM192A, resulting in its upregulation in the cytoplasm. The increased levels of circFAM192A subsequently bind to the solute carrier family 7 member 5 (SLC7A5) protein, inhibiting its degradation. SLC7A5, a member of the SLC superfamily, functions as a transporter embedded in the cell membrane, facilitating the uptake of leucine into cells (107). Evidence suggests that SLC7A5 plays a significant role in tumorigenesis through the activation of the mTOR pathway (108). Consequently, a substantial amount of undegraded SLC7A5 is retained in the membrane, which enhances leucine transport, thereby activating the mTOR pathway and ultimately promoting tumor growth.

**FIGURE 3:**
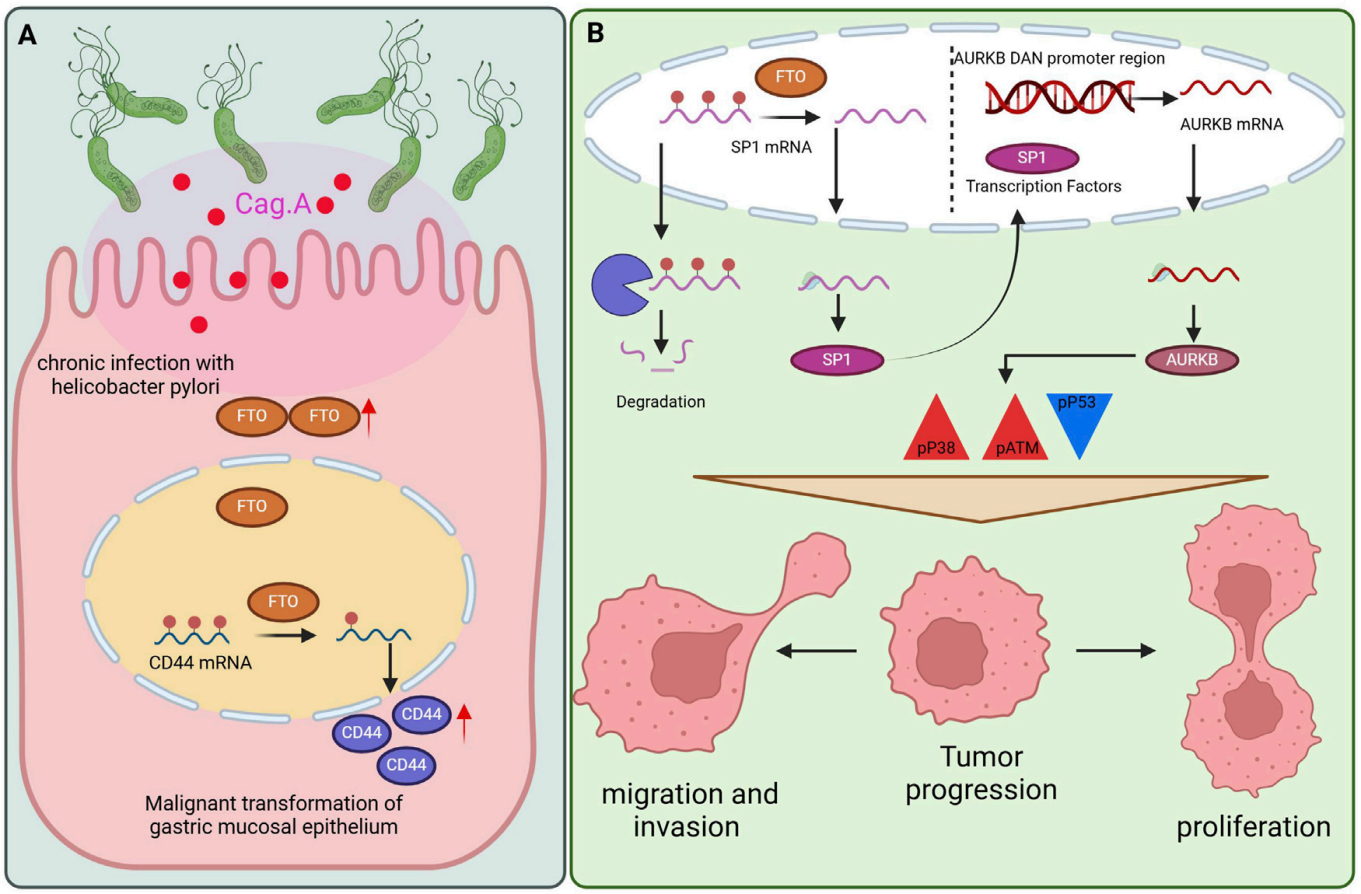
The role of FTO in GC. **(A)** The mechanism by which FTO promotes gastric carcinoma by modifying CD44mRNA methylation. **(B)** FTO can promote cell proliferation and metastasis mechanisms by regulating the SP1-AURKB-ATM axis.

FTO enhances the risk of GC by modifying CD44 mRNA methylation (109). CD44, a member of the family of cell adhesion molecules (CAMs), functions as the main receptor for hyaluronic acid (HA) and is crucial for mediating the proliferation, invasion, and migration of cells via various signal transduction pathways (110). This study suggested that the Cag A. *H. pylori*/FTO/CD44 pathway plays a role in the malignant transformation of GES-1 cells, suggesting that FTO could facilitate gastric carcinogenesis by modifying the methylation of CD44 mRNA (Figure 3A). Additionally, FTO might act as a biomarker and therapeutic target for the malignant transformation of gastric epithelial cells, providing new perspectives for the prevention and treatment of GC. Nonetheless, the exact processes involved in the control of malignant transformation within the gastric mucosa require additional exploration (109).

Another team discovered that FTO may facilitate the migration and invasion of GC cells via the ITGB1-FAK pathway (111). This research has determined that ITGB1 is a potential demethylation target of FTO (111). ITGB1 interacts with α subunits to form 12 integrin receptors, which engage with various extracellular matrix molecules, thereby activating the formation of plaque adhesion complexes and playing a critical role in cancer promotion and invasion (112). The level of phosphorylated FAK (p-FAK) decreased with FTO knockdown (FTO-KD) but could be partially reversed by ITGB1 overexpression (ITGB1-OE). These findings indicate that FTO promotes the migration and invasion of GC cells through the ITGB1-FAK pathway (111).

The degradation of Monooxygenase DBH Like 1 (MOXD1) mRNA is regulated by FTO through M^6^A modification, linking this process to unfavorable outcomes in GC and the enhancement of a malignant phenotype in GC cells (113). The MOXD1 gene is part of the monooxygenase family, which relies on copper and ascorbic acid and plays a role in various essential biological functions within living organisms (114). Under normal cellular growth conditions, this gene remains dormant or is involved only in the regulation of essential physiological processes (115). When normal cells undergo malignant transformation as a result of different factors, MOXD1 is abnormally expressed, which facilitates tumorigenesis and the progression of tumors (116). MOXD1 activates several signaling pathways, including the TGF-BETA, NOTCH, KRAS, MAPK, and JAK/STAT pathways, all of which contribute to the proliferation, migration, and invasion of cancer cells. In conclusion, FTO modulates the breakdown of MOXD1 mRNA via M^6^A modification. Elevated levels of MOXD1 expression correlate with unfavorable outcomes in GC and contribute to the development of a malignant phenotype in GC cells.

Research has shown that FTO, the primary M^6^A demethylase, increases caveolin-1 mRNA levels via demethylation. This process modulates mitochondrial fission, fusion, and metabolism, promoting the proliferation and migration of GCs (117). Caveolin-1 is a membrane protein that is abundantly expressed in adipocytes, endothelial cells, lung cells, fibroblasts, and muscle cells (118). Notably, caveolin-1 has been shown to function as both an antiapoptotic and a proapoptotic protein, acting as a tumor promoter in some contexts and a tumor inhibitor in others (119). Moreover, caveolin-1 can stimulate metastasis and serves as a prognostic marker (120). Caveolin-1 is associated with the number of mitochondria and the bioenergetic functions of various cell types (121). High levels of caveolin-1 inhibit mitochondrial function and suppress tumor activity (122). Researchers have reported that FTO depletion significantly induces mitochondrial fission and inhibits mitochondrial metabolism; however, these effects are largely reversed by caveolin-1 inhibition, indicating that the impact of FTO depletion on mitochondria is contingent upon the upregulation of caveolin-1.

A research team proposed that FTO can promote cell proliferation and metastasis by regulating the SP1-AURKB-ATM axis (123) (Figure 3B). They identified SP1 and AURKB as downstream targets of FTO, thereby elucidating the signal transduction axis involved in the progression of GC. AURKB, a critical node downstream of the FTO/SP1/AURKB pathway, can modulate the tumor microenvironment of GC and drive its malignant progression. FTO enhances the malignancy of GC cells by increasing the expression of SP1 and AURKB and by activating ATM and p38 through increased phosphorylation of these proteins, resulting in p53 inactivation. P53, a tumor suppressor, plays a crucial role in maintaining genomic stability and limiting abnormal cell proliferation. Numerous studies have indicated that p38 MAPK is instrumental in tumor cell invasion and metastasis by regulating EMT in tumor cells (124). In summary, the findings of this study highlight the potential of FTO, SP1, AURKB, and ATM as prognostic biomarkers and therapeutic targets for GC.

Moreover, MYC triggers the expression of FTO during EBV infection, potentially promoting the development of GC via the FTO-FOS-IGF2BP1/2 pathway (125). Researchers have recognized FOS as the main downstream target gene of FTO (126). In cells with EBV-associated GC (EBVaGC), overexpressed FTO removes the M^6^A modification from FOS mRNA via its demethylase function, leading to the natural degradation of FOS mRNA and a consequent decrease in its expression. This process, in turn, suppresses the migration and invasion of EBVaGC cells (125). This research revealed that IGF2BP1/2 has the ability to attach directly to a particular region of M^6^A modification within FOS mRNA. Silencing IGF2BP1/2 results in the inhibition of FOS expression and a reduction in its mRNA lifespan. In summary, the FTO-FOS-IGF2BP1/2 pathway represents a prospective treatment avenue for GC patients and will be investigated further in our upcoming research.

These findings demonstrate that FTO, as a potential biological target, may play a vital role in GC.

#### 3.1.7 ALKBH5

One study revealed that ALKBH5 can inhibit YY1 mRNA methylation, thereby negatively regulating the expression of YY1, inducing ATG4B-dependent autophagy, and influencing the proliferation and metastatic activity of GC cells (127). This study also demonstrated that YTHDF1 recognizes the methylation of YY1 mRNA to maintain its stability, resulting in increased YY1 expression, indicating that the M^6^A/YY1/ATG4B axis represents a potential therapeutic target for GC (127). YY1 is a highly conserved transcription factor that plays a crucial role in regulating a variety of genes (128). It can recruit histone acetyltransferases and deacetylases to the promoters of target genes, thereby activating or inhibiting gene expression (129). Moreover, YY1 is a key regulator of autophagy-related genes in GC. By binding to promoters, YY1 can increase PVT1 expression, further influencing autophagy through the mTOR pathway and increasing cancer invasion and adhesion activity (130). YY1 activates the ATG4B-dependent autophagy pathway by binding to the ATG4B promoter, leading to increased autophagy and progression in GC, as well as a significant increase in the level of YY1 M^6^A in GC cells. Additionally, the M^6^A-ALKBH5-YTHDF1 axis plays a critical role in the stability of YY1 mRNA. Notably, ALKBH5 reduces YY1 methylation, whereas YTHDF1 enhances the sensitivity of M^6^A methylation recognition and stabilizes mRNA, ultimately promoting YY1 expression (127).

A separate investigation revealed that ALKBH5 plays a role in mediating the M^6^A modification of circFOXP1 during the progression of GC. Additionally, circFOXP1 enhances the proliferation of GC cells by modulating the expression of SOX4 via a miR-338-3p sponge mechanism (131). Recent research has shown that the expression of circFOXP1 is significantly increased in human GC tissues compared with adjacent normal tissues, with this heightened expression linked to improved proliferation, invasion, and progression of GC cells (131). Additionally, this study identified miR-338-3p as a downstream target gene that binds to circFOXP1. CircFOXP1 therefore enhances the growth and invasion of GC cells by influencing the levels of miR-338-3p (131). Additionally, SOX4 has been identified as a target of miR-338-3p in GC (132). Recent studies suggest that circFOXP1 affects the expression of SOX4 through the regulation of miR-338-3p within the framework of GC. Notably, overexpression of ALKBH5 resulted in decreased levels of total M^6^A and circFOXP1 M^6^A in GC cells while simultaneously increasing circFOXP1 expression (131).

A recent study demonstrated that LINC00659 and YTHDF2 modulate ALKBH5-mediated JAK1 mRNA, thereby enhancing the proliferation and metastasis of GC (133). ALKBH5 interacts with JAK1 to remove the M^6^A modification from JAK1 mRNA, subsequently increasing the stability of JAK1 mRNA. JAK1, a member of the JAK family, phosphorylates STAT proteins. Upon translocation from the cytoplasm to the nucleus, phosphorylated STAT proteins activate genes that are crucial for cell survival and proliferation (134). Notably, JAK1 can phosphorylate STAT3, and the activated JAK1/STAT3 pathway is instrumental in the proliferation and metastasis of GC (135). Additionally, a previous study highlighted the interaction between JAK1 and YTHDF2 (136). The latest study revealed that the inhibition of YTHDF2 significantly increased the expression of JAK1 mRNA, whereas the overexpression of ALKBH5 diminished the binding affinity between YTHDF2 and JAK1. These findings indicate that the degradation of JAK1 mRNA is dependent on YTHDF2 and is attenuated by ALKBH5, leading to the upregulation of JAK1 expression (133). Furthermore, noncoding RNAs may function as scaffolds to facilitate interactions between mRNAs and proteins. This study revealed that LINC00659 directly binds to ALKBH5 without affecting its expression. Notably, the long-strand noncoding RNA LINC00659 enhances the formation of the ALKBH5-JAK1 complex, thereby increasing JAK1 mRNA expression in GC (133).

The M^6^A eraser ALKBH5 has been shown to downregulate the levels of NEAT1 M^6^A, and the expression of ALKBH5 is positively correlated with that of NEAT1. As NEAT1 methylation decreases, NEAT1 expression is upregulated, thereby promoting the malignant phenotype of GC (137). Dysregulation of lncRNAs is associated with the formation, progression, metastasis, and prognosis of various tumors (138). Notably, the lncRNA NEAT1 has been found to be overexpressed in multiple cancers (139), and its upregulation is positively correlated with poor overall survival outcomes (140). NEAT1 exerts its oncogenic effects through three primary mechanisms: it serves as a miRNA sponge, antagonizing the interaction between tumor suppressor gene miRNAs and their target mRNAs (139); it functions as a scaffold to interact with EZH2, enhancing the expression of downstream genes regulated by EZH2 (141); and it promotes DNA methylation by inhibiting the expression of miR-129 (142). Recent findings indicate that NEAT1 is upregulated in GC and that its overexpression can act as a scaffold to influence EZH2 expression, thereby affecting tumor invasion and metastasis (137).

These findings demonstrate that ALKBH5, as a potential biological target, may play an important role in GC.

#### 3.1.8 YTH family proteins

Previous reports indicate that YTHDF1 functions as a regulatory factor that promotes cancer development in patients with GC. YTHDF1 facilitates tumorigenesis and metastasis in GC by mediating the translation of USP14 in an M^6^A-dependent manner (143). Importantly, the expression of USP14 is positively correlated with the level of YTHDF1 and is linked to a negative prognosis in patients with GC. The inhibition of USP14 is particularly detrimental to cancer cells, as it disrupts proteasome function and leads to the accumulation of proteasome substrates (144). This study further revealed that YTHDF1 promotes USP14 protein translation in an M^6^A-dependent manner and that overexpression of USP14 can counteract the tumor-suppressive effects of YTHDF1 knockdown in GC cells. Additionally, researchers have reported that IU1, an inhibitor of USP14, can suppress cell growth and mitigate the tumor-promoting effects induced by YTHDF1 in GC cells.

A recent study demonstrated that YTHDF1 is highly expressed in response to N-methyl-N-nitrosourea (MNU) stimulation, subsequently promoting the translation of the HSPH1 protein in an M^6^A-dependent manner, which facilitates tumor cell proliferation (145). Experimental findings have indicated a strong correlation between YTHDF1 expression and MNU-induced stimulation of the M^6^A modifier (146). Prolonged exposure to low doses of MNU has been shown to induce malignant transformation through the upregulation of YTHDF1. Furthermore, the inhibition of YTHDF1 expression partially reversed the malignant transformation of the affected cells (145). HSPH1, a member of the mammalian Hsp110 family, plays a crucial role in promoting the dissociation of protein aggregates, thereby preventing the accumulation of misfolded proteins (147). In cancers of the digestive system, particularly in China, HSPH1 is overexpressed, which is correlated with a negative prognosis for affected patients (148). Furthermore, HSPH1 has the capacity to activate signaling pathways and transcription factors that are linked to the proliferation of tumor cells (149).

Among the M^6^A regulatory factors, YTHDF2 is the first to be explored and is recognized as the most effective M^6^A reader (43). A recent investigation revealed that PPP2CA might play a role in the modulation of cell proliferation, migration, and chemotherapy resistance regulated by YTHDF2. These findings indicate that focusing on the YTHDF2/PPP2CA pathway may represent a viable therapeutic approach for GC (150). Additionally, the study revealed that YTHDF2 can inhibit the progression of GC by regulating PPP2CA, demonstrating for the first time an antitumor effect in an M^6^A-independent manner. PP2Ac, which represents the catalytic subunit of PP2A and is encoded by the gene PPP2CA, is a protein that is broadly expressed and operates under tightly regulated mechanisms (151). Recognized as a tumor suppressor, alterations or functional inactivation of PP2A have been observed across a range of tumors (152). In summary, PPP2CA acts as a tumor suppressor that may be activated by PP2A-activating drugs or inhibited through antagonistic inhibitors of PP2A (153).

These findings indicate that YTH family proteins may serve as important biological targets in GC.

#### 3.1.9 IGF2BP family proteins

Research has shown that elevated expression of IGF2BP2 has been linked to unfavorable outcomes in multiple types of human cancers (154). Recent research has revealed that IGF2BP2-mediated M^6^A modification can increase the stability of CSF2 mRNA, thereby increasing CSF2 expression. The increased expression of CSF2 results in the ubiquitination of Notch1, subsequently inactivating Notch signal transduction. Together, these molecular changes alter the phenotype and functionality of mesenchymal stem cells (MSCs), indicating that the IGF2BP2/CSF2/Notch1 pathway plays a role in enhancing tumor-associated traits via epigenetic modulation of the TME (154). MSCs are well recognized for their significant role in shaping the TME and can be reprogrammed by GC cells to promote a tumor phenotype through epigenetic mechanisms (155). CSF2 is recognized for its ability to draw in and support the survival of microglia and macrophages within the glioblastoma microenvironment, thereby facilitating tumor polarization (156). The Notch signaling pathway is crucial for regulating the characteristics and capabilities of MSCs; for example, the activation of this pathway has been shown to improve the migratory abilities of MSCs (157). In a recent study, CSF2 was shown to trigger ubiquitination and subsequent downregulation of Notch1 in MSCs, thus fostering the tumor phenotype and functionality. These findings suggest that Notch1 is pivotal in the reprogramming of cancer-associated MSCs.

### 3.2 The role of M^5^C modification in GC

5-Methylcytosine is a type of RNA modification that has garnered increasing attention because of its ability to dynamically regulate various biological functions through associated regulatory factors. Numerous studies have confirmed that the dynamic modification of M^5^C and its regulatory factors plays a significant role in a range of physiological and pathological processes, including RNA stability, gene expression, and protein synthesis. M^5^C is recognized as crucial for RNA expression, alternative splicing, transport, stability, and translation (49). Furthermore, imbalances in M^5^C RNA modifications have been implicated in various human malignant tumors, suggesting that M^5^C modification holds considerable potential for cancer treatment.

#### 3.2.1 NSUN2

A previous study demonstrated that the RNA methyltransferase NSUN2 is highly expressed in GC and functions as a prognostic biomarker linked to unfavorable outcomes (158). This investigation further revealed that reducing NSUN2 expression suppresses the growth and spread of GC cells *in vitro*, whereas increased NSUN2 levels facilitate these activities. It has been discovered that NSUN2 can directly interact with SUMO-2/3 (158). SUMOylation stands out as a crucial regulatory posttranslational modification that can impact protein stability, protein localization within the cell, and the functional characteristics of substrate proteins (159). This study posits that NSUN2 is capable of catalyzing the M^5^C modification of PIK3R1 and PCYT1A, thus influencing their expression and fostering the development of GC. Consequently, the SUMOylation-NSUN2-M^5^C axis may represent a novel diagnostic and therapeutic target for GC and pancancer therapy (158).

#### 3.2.2 NSUN5

Increased expression of NSUN5 in GC plays a significant role in regulating cell proliferation and migration via the WNT/β-catenin pathway while simultaneously inhibiting CD8^+^ T-cell infiltration within the immune microenvironment of GC (160). These findings indicate that NSUN5 is significantly upregulated in GC and is correlated with poor survival and prognosis for patients, thus establishing NSUN5 as a key driver gene in the promotion of GC. Furthermore, this study revealed a significant relationship between NSUN5 and the WNT signaling pathway, confirming the influence of NSUN5 on this pathway through the pivotal protein β-catenin. The WNT pathway is essential for various cellular functions, including development and differentiation (161). It is activated by upstream signals, leading to β-catenin dephosphorylation and subsequent nuclear translocation for transcriptional regulation, which contributes to the immunosuppressive microenvironment observed in cancer cells (162). Recent findings suggest that NSUN5 can inhibit the infiltration of CD8^+^ T cells within the immune microenvironment of GC, promoting immune escape (160). In summary, NSUN5 not only accelerates the proliferation and migration of GC cells through the WNT pathway but also diminishes the immune microenvironment of CD8^+^ T cells, contributing to the malignant progression of GC.

#### 3.2.3 YBX1

Studies have indicated that YBX1 is hyperactive in GC, and its knockdown suppresses the proliferation of GC cells. Furthermore, the same researchers observed significant upregulation of HOXC-AS3 in GC tissues and proposed that this long non-coding RNA plays a critical role in gastric tumorigenesis. The oncogenic function of HOXC-AS3 is partially mediated through its interaction with YBX1 (163). Specifically, HOXC-AS3 binds to YBX1, leading to transcriptional regulation of multiple genes associated with cell proliferation and migration—such as MMP7, WNT10B, and HDAC5—in gastric cancer cells, thereby promoting malignant behaviors (163). In summary, aberrant histone modification-mediated activation of the novel lncRNA HOXC-AS3 promotes GC cell proliferation and migration via interaction with YBX1 and subsequent transcriptional activation of a broad spectrum of downstream genes. These findings elucidate the role of HOXC-AS3 in GC tumorigenesis and may inform strategies targeting HOXC-AS3 as a potential biomarker and therapeutic target for GC patients.

### 3.3 The role of M^1^A modification in GC

In the current surge of epigenetic research, the field of RNA modification has garnered significant attention, particularly regarding M^6^A and M^5^C modifications, which have emerged as focal points for researchers. In contrast, few studies have focused on the mechanisms of M^1^A modification in GC. Current research in this area is still in its infancy, with only preliminary investigations providing a glimpse into its potential implications. In the future, there is considerable promise for researchers to delve into the intricate mechanisms of M^1^A modification in GC, including proliferation, apoptosis, migration, and invasion, as well as their interactions with diverse cell types in the TME.

### 3.4 The role of M^7^G in gastric cancer

#### 3.4.1 METTL1 and WDR4

METTL1, a key regulator of M^7^G modification, is significantly upregulated in hepatocellular carcinoma and associated with poor patient prognosis (164). It is also known to exhibit oncogenic activity through the PTEN/AKT signaling pathway. Studies indicate that METTL1 knockdown suppresses the proliferation, migration, and invasion capabilities of GC cells. This finding was corroborated by observations of slower tumor growth in METTL1-knockdown cells, suggesting that METTL1 depletion attenuates GC proliferation *in vivo* and implicating METTL1 in promoting GC progression (165). Furthermore, one study demonstrated that WDR4 overexpression enhances the proliferative and metastatic potential of HGC-27 and MKN1 cells. Conversely, WDR4 knockdown was shown to reduce cell growth, metastatic capacity, and colony-forming ability, indicating a promotive role for WDR4 in GC progression (165).

Substantial experimental evidence from this study revealed that METTL1 enhances the activity of mitochondrial Electron Transport Chain (ETC) Complex II in GC cells. The function of the mitochondrial, ETC is closely linked to ATP generation, oxidative phosphorylation (OXPHOS), and the production of tricarboxylic acid (TCA) cycle metabolites (166). The researchers identified SDHAF4 as a critical METTL1 target responsible for enhancing, ETC Complex II activity in GC (165). Additionally, SDHAF4 knockdown rescued the METTL1 overexpression-induced decrease in ubiquitination levels of SDHA and SDHB, demonstrating that METTL1-mediated suppression of SDHA and SDHB ubiquitination is dependent on SDHAF4. These findings suggest that SDHAF4 acts as a potential oncogene and a key downstream effector of METTL1 in GC. Ultimately, the METTL1/M^7^G/SDHAF4/SDHA/SDHB/ETC Complex II axis may represent a potential therapeutic target for GC.

## 4 Therapeutic prospects and challenges of targeting RNA methylation

The intricate involvement of RNA methylation modifiers in GC pathogenesis positions them as attractive therapeutic targets. The development of small-molecule inhibitors against these epigenetic enzymes represents a burgeoning frontier in oncology research, with the potential to overcome therapy resistance and improve patient outcomes.

Substantial progress has been made in targeting the m6A machinery. The METTL3-specific inhibitor STM2457 has demonstrated preclinical efficacy not only in directly suppressing tumor growth but also in modulating the tumor immune microenvironment. In GC models, STM2457 was shown to restore PD-L1 expression and enhance the cytotoxic function of CD8^+^ T cells, presenting a promising combinatorial strategy to augment immunotherapy response (167). Concurrently, inhibitors targeting the demethylases FTO and ALKBH5 are under active investigation. For instance, MV1035, initially identified as a sodium channel blocker, effectively suppresses ALKBH5 activity through off-target interactions, impairing the migration and invasion of cancer cells (168). Furthermore, the specific ALKBH5 inhibitor ALK-04 has been shown to synergize with anti-PD-1 immunotherapy in melanoma, providing a compelling rationale for testing such combinations in GC (169).

Despite this promising outlook, several formidable challenges must be addressed. The ubiquitous expression and fundamental biological roles of many RNA-modifying enzymes raise concerns about on-target toxicity, necessitating the development of highly selective inhibitors or targeted delivery systems. The context-dependent dual roles of regulators like METTL14 and ALKBH5 complicate patient stratification and predictive biomarkers are urgently needed to identify populations most likely to benefit. Moreover, the current reliance on preclinical models and the significant gap between their results and clinical efficacy in humans underscores the need for more sophisticated experimental systems. Finally, a deeper understanding of the functional crosstalk between different RNA modifications (e.g., m^6^A, m^5^C, m^7^G) will be crucial for designing effective multi-targeted or sequential therapeutic regimens.

## 5 Conclusion

In summary, this review synthesizes the rapidly advancing field of RNA methylation in GC, highlighting its central role as a master regulator of gene expression that governs virtually every aspect of GC pathogenesis, including cell proliferation, apoptosis, metastasis, and therapy resistance. We have delineated the distinct and overlapping functions of key modifications such as m^6^A, m^5^C, m^1^A, and m^7^G, and critically evaluated their associated writers, erasers, and readers. We have identified that m6A and m5C play complementary yet distinct roles in driving gastric cancer progression. m6A acts as a rapid, dynamic, and multifunctional regulatory hub that finely tunes the transient equilibrium between oncogenic and tumor-suppressive signaling pathways. In contrast, m5C functions more as a robust post-transcriptional enhancer, stabilizing the mRNA of key oncogenes to sustain the malignant phenotype of tumors. This distinction is crucial for future research and therapeutic development. Targeting the m6A pathway may require careful consideration of its complex contextual effects, with therapeutic strategies potentially focusing on inhibiting pro-tumor writers or activating tumor-suppressive erasers in specific tumor microenvironments. For m5C, the approach could be more straightforward, primarily centered on inhibiting its writers to disrupt the persistent enhancement of the oncogenic network. Integrating the regulatory networks of these two modifications will provide a solid theoretical foundation for developing more precise epi-transcriptomic therapies for gastric cancer. The landscape of RNA methylation in GC is characterized by both complexity and promise. While regulators like METTL3 and FTO largely drive oncogenesis, others, most notably METTL14, exert potent tumor-suppressive effects, underscoring the critical importance of context-specific understanding. The associated molecules hold significant potential as diagnostic and prognostic biomarkers, and perhaps more importantly, as novel therapeutic targets. Although the mechanistic understanding of M^1^A modification in GC remains in its infancy, it represents a fertile ground for future discovery. Looking forward, the translation of these fundamental insights into clinical practice hinges on overcoming the challenges of drug development and patient stratification. The integration of RNA methylome analysis into clinical diagnostics, combined with the development of targeted epigenetic therapies, is poised to open new avenues for the precise and effective treatment of gastric cancer, ultimately offering hope for improved patient survival.

## Glossary

GC: Gastric cancer
M^6^A: N6-methyladenosine
M^5^C: 5-methylcytidine
M^1^A: N1-methyladenosine
M^7^G: N7-methylguanosine
M^3^C: 3-methylcytidine
Ψ: pseudouridine
ncRNAs: noncoding RNAs
mRNA: messenger RNA
tRNA: transfer RNA
rRNA: ribosomal RNA
snRNA: small nuclear RNA
snoRNA: small nucleolar RNA
lincRNAs: long intergenic noncoding RNAs
circRNAs: circular RNAs
METTL3: methyltransferase-like 3
METTL14: methyltransferase-like 14
METTL1: methyltransferase-like 1
WDR4: WD repeat domain 4
WTAP: nephroblastoma-1 associated protein
SAM: S-adenosylmethionine
SAH: S-adenosylhomocysteine
RNM15: RNA-binding motif protein 15
MTC: methyltransferase complex
ZC3H13: zinc finger CCCH-type containing 13
LC: low-complexity
METTL16: Methyltransferase-like 16
FTO: fat mass and obesity-related protein
ALKBH5: AlkB homolog 5
DSBH: double-stranded beta-helix
PKMYT1: protein kinase, membrane associated tyrosine/threonine 1
YTHDF: YTH domain family
HNRNP: heterogeneous ribonucleoprotein
IGF2BP: insulin-like growth factor 2 mRNA-binding proteins
FMRP: fragile X mental retardation protein
SRSF2: splicing factor 2
CSD: cold shock domain
RBPs: RNA binding proteins
TRMT6: tRNA Methyltransferase 6 Non-Catalytic Subunit
HCC: hepatocellular carcinoma
HBXIP: Hepatitis B X-interacting protein
SOCS: suppressor of cytokine signaling
EMT: epithelial‒mesenchymal transformation
ZMYM1: zinc finger MYM type 1
ANGPTL3: angiogenin-like 3
STAD: STomach ADenocarcinoma
RTKs: receptor tyrosine kinases
GPCRs: G protein-coupled receptors
TCGA: The Cancer Genome Atlas
SLC7A5: solute carrier family 7 member 5
CAMs: cell adhesion molecules
HA: hyaluronic acid
p-FAK: phosphorylated FAK
FTO-KD: FTO knockdown
ITGB1-OE: ITGB1 overexpression
MOXD1: Monooxygenase DBH Like 1
EBVaGC: EBV-associated gastric cancer
EBV: Epstein–Barr virus
USP14: ubiquitin-specific protease 14
MSCs: mesenchymal stem cells
GBM: pleomorphic glioblastoma
ACLY: ATP-citrate lyase
